# Spatial Characteristics, Sources of Volatile Organic Compounds and Effects on O_3_ Formation in Summer in Taiyuan, China

**DOI:** 10.3390/toxics14030220

**Published:** 2026-03-04

**Authors:** Lili Guo, Tianyu Gao, Bingxi Wang, Yang Cui, Qiusheng He, Zhentao Wang, Xiaojing Hu, Xinming Wang

**Affiliations:** 1 School of Materials Science and Engineering, Taiyuan University of Science and Technology, Taiyuan 030024, China; guolili1@tyust.edu.cn; 2 Shanxi Key Laboratory of Coordinated Management and Control for Environmental Quality, School of Environment and Resources, Taiyuan University of Science and Technology, Taiyuan 030024, China; s202323111111@stu.tyust.edu.cn (T.G.); wbxtyust198@163.com (B.W.); wangzt@tyust.edu.cn (Z.W.); s202323111112@stu.tyust.edu.cn (X.H.); 3 State Key Laboratory of Organic Geochemistry, Guangzhou Institute of Geochemistry, Chinese Academy of Sciences, Guangzhou 510640, China; wangxm@gig.ac.cn

**Keywords:** VOCs, OVOCs, spatial heterogeneity, ozone formation potential, source apportionment

## Abstract

Many previous studies on volatile organic compounds (VOCs) have focused on Photochemical Assessment Monitoring Station (PAMS) VOCs at a single site, yet there is limited understanding of the spatial heterogeneity of both PAMS VOCs and oxygenated VOCs (OVOCs) across multiple functional zones at the city scale. To better understand the characteristics, sources and the effects of VOCs on O_3_, we conducted simultaneous measurements of 71 VOCs (57 PAMS VOCs and 14 OVOCs) at three urban sites (Taoyuan, TY; Jinyuan, JY; Xiaodian, XD) and one suburban site (Shanglan, SL) in Taiyuan, a heavily industrialized city in northern China, during the summertime of 2022 and 2023. Total VOCs (TVOCs) concentrations were comparable at SL (21.9 ± 7.7 ppbv) and JY (21.9 ± 8.7 ppbv), but higher than those at TY (20.3 ± 6.3 ppbv) and XD (19.5 ± 6.4 ppbv). OVOCs were the dominant component at all sites, accounting for over 60% of TVOCs, with formaldehyde as the most abundant species. Ozone formation potential (OFP) followed the order of SL (119.6 ± 47.7 ppbv) > JY (112.0 ± 58.2 ppbv) > TY (100.4 ± 34.2 ppbv) > XD (97.1 ± 34.1 ppbv), with OVOCs contributing over 75% to the total OFP. Positive matrix factorization (PMF) resolved seven sources, with secondary formation as the largest contributor at all sites (24.6–32.5% of TVOCs, 30.5–37.0% of OFP). The second-largest source of VOCs and OFP exhibited a systematic spatial gradient: biogenic sources at SL (22.0%, 28.9%), gasoline vehicle exhausts at TY (22.5%, 21.8%), coking sources at JY (23.9%, 22.8%), and combustion sources at XD (23.6%, 26.0%). The lack of OVOCs could lead to an overestimation of primary sources and an underestimation of photochemical processing in source apportionment studies. These findings demonstrate that zone-specific measures should be complemented by regional precursor reductions for effective O_3_ mitigation in Taiyuan.

## 1. Introduction

Tropospheric O_3_ is a secondary pollutant mainly generated by volatile organic compounds (VOCs) and nitrogen oxides (NOx) through a series of photochemical reactions under sunlight [1]. Due to the adverse effects, O_3_ pollution has attracted widespread attention in recent years [2,3]. Elevated O_3_ concentrations could enter plants through stomata, damaging photosynthesis and reducing carbon fixation, which causes significant crop yield losses and decreases forest timber production [4,5]. O_3_ could also pose a threat to human health by penetrating the respiratory tract, triggering inflammation, and exacerbating asthma and cardiovascular diseases [6]. In China, O_3_ has shown an increasing trend, with the 90th percentile of the daily maximum 8 h average concentration of O_3_ rising at a rate of 2.19 ppb yr^−1^ during the period 2014~2020 [7]. Despite the nonlinear relationship between O_3_ formation and its precursors (VOCs, NOx), a lot of previous studies have found that the formation of O_3_ in the urban area of many Chinese cities was VOC-limited [8,9,10], and a reduction in VOCs could decrease the O_3_ concentration. Hence, it is necessary to carry out refined studies on characteristics and sources of VOCs to make scientific control measures to abate O_3_ pollution.

Currently, extensive research has been conducted on VOC pollution in major Chinese city clusters, including the Beijing–Tianjin–Hebei region [9,10,11], the Pearl River Delta [12], and the Yangtze River Delta [13,14]. These studies have provided valuable insights into the composition, sources, and O_3_ formation potential of VOCs. However, most previous studies have focused on Photochemical Assessment Monitoring Station (PAMS) VOCs at a single monitoring site, including alkanes, alkenes, aromatic hydrocarbons and alkynes [15,16,17], potentially underestimating the role of oxygenated VOCs (OVOCs) in O_3_ formation. OVOCs, including aldehydes and ketones, are emitted directly or formed secondarily through photochemical processes [18]. OVOCs are increasingly recognized as critical contributors to ozone formation potential (OFP), accounting for 30–70% of total OFP in some Chinese cities during summer [19,20,21]. Formaldehyde alone can contribute 29–62% to total OFP depending on the region [22,23,24]. Furthermore, the spatial heterogeneity of VOC sources within cities remains poorly understood. Due to differences in land use, industrial distribution, and traffic patterns, VOCs concentrations and sources can vary significantly across different functional zones [19,25,26,27]. Single-site studies, while informative, cannot capture this spatial variability, leading to potentially oversimplified control recommendations. Simultaneous multi-site measurements that include both PAMS VOCs and OVOCs are therefore needed to provide a more complete picture of urban VOCs pollution characteristics and sources.

Taiyuan, the capital city of Shanxi province, is an important energy base in China and a typical industrial city in the Fenwei Plain dominated by the coking and steel industries. In recent years, O_3_ has become the primary pollutant affecting air quality in summer, and the 90th percentile of the daily maximum 8 h average concentration of O_3_ in Taiyuan exceeded the national standards (160 µg/m^3^), reaching 186 µg/m^3^, 186 µg/m^3^ and 192 µg/m^3^ in 2019, 2020 and 2021, respectively. Based on O_3_ sensitivity analysis derived from satellite and ground-based observations, Taiyuan City exhibits O_3_ formation characteristics predominantly within the VOC-limited regime [28,29]. Previous studies in Taiyuan have either focused on a single site [3,18] or measured only PAMS VOCs [2,3]; the simultaneous measurements of both PAMS VOCs and OVOCs across multiple functional zones were limited. Given the above reasons, this study conducted simultaneous measurements of 57 PAMS VOCs and 14 OVOCs at three urban sites (Taoyuan, Jinyuan, and Xiaodian) and one suburban site (Shanglan) in Taiyuan during the summertime of 2022 and 2023. The specific objectives were to (1) characterize the spatial distribution of VOCs across different functional zones, (2) quantify the contribution of OVOCs to O_3_ formation potential, and (3) apportion VOC sources using positive matrix factorization (PMF) and compare the results with previous single-site, PAMS VOC-only studies. These findings allowed us to reveal the spatial heterogeneity of VOC sources and their implications for zone-specific O_3_ control in this region.

## 2. Materials and Methods

### 2.1. Sampling Sites

To better explore the spatial characteristics of VOCs, three urban sites, Taoyuan (TY), Jinyuan (JY), Xiaodian (XD), and a suburban site, Shanglan (SL), were selected in Taiyuan. The sampling heights at TY, JY XD and SL were 15 m, 18 m, 17 m and 12 m above the ground, respectively. These heights were selected based on the technical specifications on manual methods for ambient air quality monitoring (HJ 194 2017) [30] and ambient air quality standards (GB 3095-2012) [31]. Meanwhile, the sampling height effectively minimizes interference from near-ground pollution sources and captures well-mixed air masses representative of the city scale, making it suitable for comparing the spatial distribution of VOCs across different sites.

**Figure 1:**
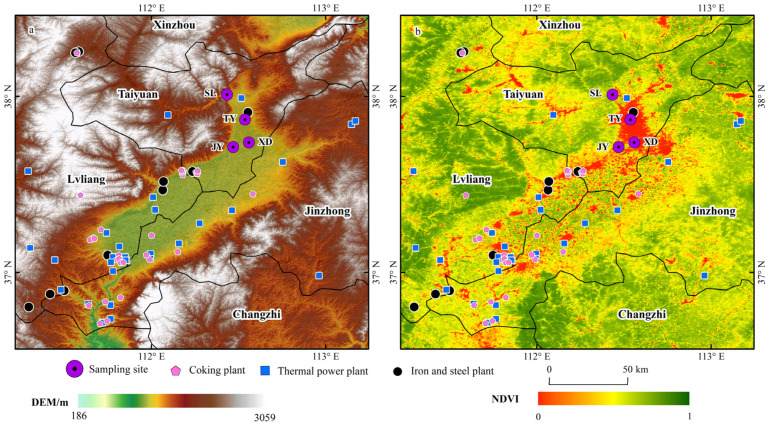
Geographical distribution of four sampling sites and major emission sources in Taiyuan Basin. (**a**) DEM (Digital Elevation Model), indicating elevation patterns; (**b**) NDVI (Normalized Difference Vegetation Index), indicating vegetation distribution. Note: NDVI is used to represent vegetation cover conditions in the study area, with higher values indicating denser vegetation.

In terms of geographical distribution, Taiyuan is located in the northern area of Taiyuan Basin (Figure 1a). The industrial sources in the southern area of Taiyuan Basin could affect the VOCs in Taiyuan under southerly wind conditions; thus, the detailed locations of the four sampling sites and major emission sources in Taiyuan Basin are shown in Figure 1. TY (37.87° N, 112.54° E) is located downtown, which is a typical commercial and residential mixed area. The site is approximately 1 km away from the main trunk traffic road (Binghe Road) and about 5 km from a large steel industrial area to the northeast, which includes steelmaking and coking processes. Taiyuan No. 2 Thermal Power Plant is located approximately 12 km north of the TY site. The JY (37.71° N, 112.47° E) site is situated in southwestern Taiyuan, approximately 20 km away from the Qingxu industrial park in the southwest direction, which contains three coke plants and several coal chemical factories. XD (37.74° N, 112.56° E) lies in southeastern Taiyuan and is encircled by four main roads characterized by heavy traffic. A freeway entrance is located approximately 1 km northwest of XD. The SL site (38.01° N, 112.43° E) is situated in the northern suburban area of Taiyuan, adjacent to Erlong Mountain and Juewei Mountain. Compared to the other three urban sites, the vegetation emissions at the SL site are relatively strong. Detailed information on the surroundings of the sampling sites is shown in Figure S1.

### 2.2. Sampling and Analysis

Since the O_3_ exceedance events in Taiyuan mainly occur in summer [32], with peak values appearing between 14:00 and 15:00 (Figure S2), PAMS VOC and OVOC samples were synchronously collected from June to August in 2022 and 2023 in this study. Samples were conducted every 6 days, with a 3 h sampling duration from 12:00 to 15:00. In total, 30 valid samples were obtained per site (SL, TY, JY, and XD), amounting to 120 samples across all sites. Additionally, a parallel sample and a blank sample were taken at each site every month, with the relative deviation for each VOC concentration in the parallel samples ≤ 20% and all VOCs in the blank samples below the method detection limit (MDL). The collection of samples was postponed by one day when there was precipitation.

Atmospheric PAMS VOCs samples were collected by 3.2L stainless steel Summa canisters (Entech Instruments, Simi Valley, CA, USA) with a constant flow integrator sampler (39-CS1200ES3, Entech Instruments, Simi Valley, CA, USA). Before sampling, the Summa canisters were repeatedly cleaned using high-purity nitrogen. The sample was analyzed by an atmospheric concentrator (Nutech 8900DS, Nutech Instruments, Dallas, TX, USA) and gas chromatography–mass spectrometry (GC-MS) (Agilent 8890A/5977B, Agilent Technologies, Santa Clara, CA, USA). A total of 58 VOCs were qualitatively and quantitatively determined using PAMS (Photochemical Assessment Monitoring Stations) and TO-15 (U.S. EPA Method TO-15) as the standard gas mixture, including 29 alkanes, 10 alkenes, 17 aromatics, acetylene and 1 OVOC (methyl tert-butyl ether, MTBE). The correlation coefficients of calibration curves for all VOC species were higher than 0.99, and the relative standard deviations of the relative response factors were less than 30%. The detailed instrumental analysis procedure can be found in our previous studies [3].

OVOC compounds were collected using silica gel sampling tubes (DNPH-silica, Waters Corporation, Milford, MA, USA) coated with 2,4-dinitrophenylhydrazine (DNPH). To prevent the impact of ozone in the atmosphere on sample collection, an ozone removal tube containing potassium iodide (CNW Technologies GmbH, Düsseldorf, Germany) was used prior to the DNPH-silica. When the gas flows through the DNPH-silica during the sampling process, OVOCs undergo a derivatization reaction with 2,4-dinitrophenylhydrazine to form hydrazine derivatives. The sampling flow rate was 1 L/min, and the sampling duration lasted for 3 h. The DNPH-silica was eluted with acetonitrile, and then the eluate was injected into a high-performance liquid chromatography (HPLC, Agilent 1260) equipped with an ultraviolet detector. The detailed liquid chromatography analysis conditions were described in our previous studies [18,24]. Each carbonyl compound was qualitatively identified by retention times and quantitatively determined by peak area. The standard curve of individual carbonyl was established based on the external standard method, with correlation coefficients higher than 0.999. In this study, a total of 13 OVOCs were detected, including formaldehyde, acetaldehyde, acetone, acrolein, propionaldehyde, crotonaldehyde, butanone, methacrolein (MACR), butyraldehyde, benzaldehyde, valeraldehyde, m/p-tolualdehyde and hexaldehyde.

During the sampling period, meteorological data (wind speed (WS) and wind direction (WD)) were obtained from the Taiyuan Environmental Monitoring Center of Shanxi Province, with a time resolution of 1 h. A wind rose diagram during the sampling period at SL, TY, JY and XD is shown in Figure S3. The prevailing winds were dominated by westerly/southwesterly directions at SL and TY, and by southerly directions at JY and XD, respectively. The influence of WS and WD on VOCs primarily involves the dilution of local emissions and the regional transport of pollutants from upwind source areas [3,18].

### 2.3. Ozone Formation Potential (OFP)

Given that the O_3_ formation sensitivity in our study region is characterized by a VOC-limited regime [28,29], the primary aim of this study is to identify the key VOC species that drive O_3_ production. This was achieved by calculating the OFP for each species, a metric that quantifies their relative contribution to O_3_ formation. OFP values were calculated using the following equation [33].

(1) $${OFP}_{i}={\;VOC}_{i}\;\times \;{MIR}_{i}$$

where OFP_i_ is the ozone formation potential of the ith species in ppbv and VOC_i_ is the concentration of the ith VOC compound in ppbv. MIR is the maximum incremental reaction of the VOC species [34].

### 2.4. Positive Matrix Factorization (PMF) Model

PMF models have been widely used for source apportionments of VOCs across various cities and regions around the world [19,25,26,27]. The US EPA PMF (version 5.0) was used to apportion the sources of VOCs in this study and a detailed introduction can be found in our previous studies [2,3,24]. In brief, the PMF model decomposes the VOCs dataset into two matrices: the source profiles and source contributions [33]. The selection of VOC species follows these principles: (1) species with a signal-to-noise ratio (S/N) below 0.2 were excluded; (2) source tracers were retained, such as isoprene, a marker for biogenic sources. Based on the above principles, 24 VOC species were selected for PMF data in this study, including 3 alkenes, 5 aromatics, 10 alkanes, 1 acetylene and 5 OVOCs. In addition, the Qtrue/Qexp ratio in our study was 0.94. As a value below 1.5 is generally considered reasonable [35], this indicates the stability of our PMF solution. Since the four sites were located within the same city, this study assumed the VOC source profiles of the four sites were consistent. To ensure robust PMF results with sufficient sample data, VOC samples from all four sites were pooled for source apportionment. Two input files were required in the PMF 5.0, one with the concentrations of observed VOC species and the other with corresponding uncertainties. The uncertainties of the input datasets were calculated using the following equation:

(2) $$Unc\;=\;\frac{5}{6}\;\times \;MDL$$

(3) $$Unc=\sqrt{{\left(Error\;Fraction\;\times \;Concentration\right)}^{2}+{(0.5\;\times \;MDL)}^{2}}$$

Here, Equation (2) was used if the concentration of the compound was less than the corresponding method detection limit (MDL), and Equation (3) was adopted if the concentration was higher than MDL. The MDL was shown in Table S1. The relevant research set the error fraction value at 20% [2]. To obtain a reasonable solution, 4 to 9 factors were explored in this study. Finally, a 7-factor solution was considered the optimum solution. More than 75% of the VOC species with correlation coefficients (R^2^) between observed and modeled values of each species from PMF were higher than 0.70 (Table S2). Displacement (DISP) and bootstrap (BS) analysis of error estimation in the PMF model were used to evaluate the stability of the base run solution. The uncertainties of the PMF results for the 7-factor solution were evaluated by DISP and BS. The absence of factor swapping in the DISP results confirmed the reliability of the solution. After 100 BS runs, more than 80% of the BS runs could be mapped to base runs (Table S3).

## 3. Results and Discussion

### 3.1. Chemical Compositions

**Figure 2:**
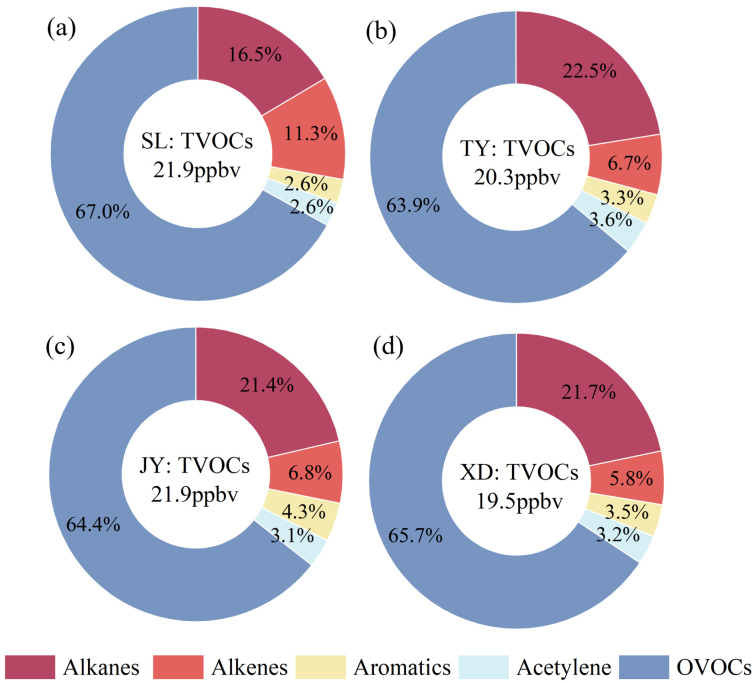
Contributions of the VOC groups at (**a**) SL, (**b**) TY, (**c**) JY and (**d**) XD.

**Figure 3:**
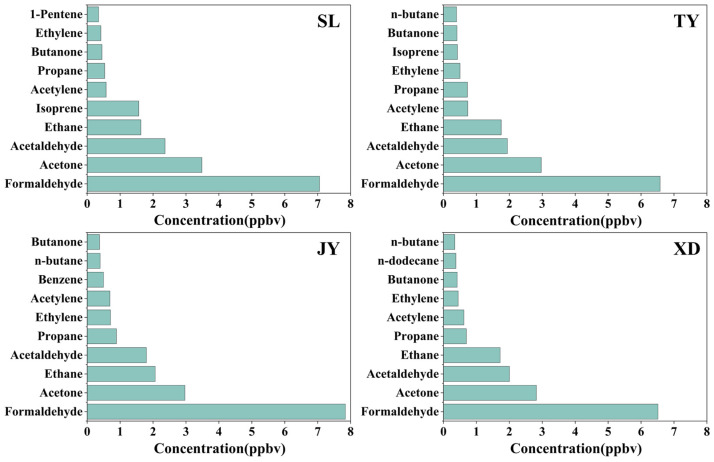
Concentration of the top 10 VOC species at SL, TY, JY and XD.

In this study, 71 VOCs were analyzed, including 29 alkanes, 10 alkenes,1 alkyne, 17 aromatics and 14 OVOCs. The average concentration of total VOCs (TVOCs) at SL (21.9 ± 7.7 ppbv) was comparable to that at JY (21.9 ± 8.7 ppbv), but higher than those at TY (20.3 ± 6.3 pppbv) and XD (19.5 ± 6.4 ppbv). The contributions of five main organic classes to TVOCs at the four sites are shown in Figure 2. OVOCs (63.9–67.0%) were the dominant compounds at four sites, followed by alkanes (16.5–22.5%), alkenes (5.8–11.3%), aromatics (2.6–4.3%) and acetylene (2.6–3.6%). The OVOCs’ dominance observed in Taiyuan (>60%) is notably higher than in many other Chinese cities. For instance, in an urban site in Beijing, OVOCs contributed 40.1% to TVOCs, followed by alkanes (39.3%). In Zibo, a typical industrial city in Shandong province in China, alkanes (33.3–51.5%) and OVOCs (30.0–37.8%) at five representative sites (urban, suburban, industrial, upwind and downwind stations) were the two dominant VOC groups. Compared with the previous study in Taiyuan [3], Wang et al. found that alkanes (56.3%) were the most abundant VOC group at the JY site in the summer of 2021, with OVOCs not included in their study. Variations in industrial structure, emission intensity, and sampling period could lead to differences in the composition and proportions of VOCs among different cities. It should be noted that sampling in this study was conducted from 12:00 to 15:00, a period typically characterized by peak daily photochemical activity. This likely explains the observed OVOC dominance, which is consistent with the study in Beijing reporting elevated OVOC levels during periods of peak photochemical activity [36]. As for VOC species, the concentrations of the top 10 VOCs at four sites accounted for 81.2%~84.1% of TVOCs. Formaldehyde, acetone, acetaldehyde and ethane were the four most abundant species at four sites (Figure 3), accounting for 65.4%~67.0% of TVOCs. Especially, the average concentrations of formaldehyde (6.5–7.8 ppbv) at four sites were significantly higher than those of acetone (2.8–3.5 ppbv), acetaldehyde (1.8–2.4 ppbv) and ethane (1.6–2.1 ppbv). The dominance of formaldehyde in Taiyuan is consistent with the observations in other Chinese cities, such as Jinan, Beijing, Chongqing and Zibo [20,37,38,39]. The detailed concentrations of individual VOC species at four sites can be found in Table S4.

The contributions of OVOCs to TVOCs presented the order of SL (67.0%) > XD (65.7%) > JY (64.4%) > TY (63.9%). OVOCs originate from complex and diverse sources, including primary anthropogenic sources (e.g., vehicle exhaust, industries) and secondary formation via photochemical reactions [18]. The highest OVOCs contribution observed at the SL site can be primarily attributed to the photochemical aging of air masses and enhanced secondary formation. As a suburban background site located downwind of the main urban area, SL is more influenced by aged air masses, where primary VOCs have undergone photochemical oxidation, leading to the secondary formation of OVOCs [27]. In addition, rapid photochemical oxidation of reactive biogenic VOCs at the SL site, particularly the high proportion of isoprene, also could elevate the OVOCs fraction [40]. Among OVOC species, formaldehyde, acetone and acetaldehyde were the three dominant species, collectively contributing 87.5~88.6% to OVOCs at four sites, which was in line with the results reported in Beijing (86.4%) [38] and Zhengzhou (91.7%) [41]. The second-largest VOC group was alkanes, accounting for 16.5%, 22.5%, 21.4% and 21.7% at SL, TY, JY and XD, respectively. The highest contribution of alkanes was observed at TY due to the stronger contributions of ethane, isopentane and C6~C8 branched alkanes, which were predominantly emitted from light-duty gasoline vehicles [42]. The main roads with heavy traffic flow near the TY site could result in higher fractions of alkanes compared to the other three sites. As for alkenes, the contributions to TVOCs were in the order of SL (11.3%) > JY (6.8%) > TY (6.7%) > XD (5.8%). The highest contribution of alkenes at SL was mainly due to the higher contribution of isoprene, which was mainly emitted from biogenic sources [2]. The contribution of isoprene to alkenes at SL (63.1%) was also much higher than those at TY (31.4%), XD (26.2%) and JY (18.9%), which was similar to the findings in some typical background sites in China [43]. The aromatics accounted for 2.6%, 3.3%, 4.3% and 3.5% to TVOCs at SL, TY, JY and XD, respectively. As seen from Table S4, benzene was the most abundant aromatic compound at four sites, and the average concentration at JY (0.5 ± 1.0 ppbv) was much higher than that at XD (0.3 ± 0.5 ppbv), TY (0.2 ± 0.2 ppbv) and SL (0.2 ± 0.2 ppbv). Additionally, the average concentration of ethylene at JY (0.7 ± 1.0 ppbv) was also higher than TY (0.5 ± 0.5 ppbv), XD (0.5 ± 0.6 ppbv) and SL (0.4 ± 0.4 ppbv). Our previous studies have indicated that ethylene and benzene could be regarded as reliable tracers of a coking source [2,3]. The highest contribution of aromatics at JY could be largely ascribed to the influence of the coking plants in south area of Taiyuan. Acetylene typically originated from combustion sources and contributed 2.6%, 3.6%, 3.1% and 3.2% to TVOCs at SL, TY, JY and XD sites, respectively. Overall, the SL site, characterized as a suburban background location with minimal direct anthropogenic emissions, exhibited the lowest proportions of alkanes, aromatics, and acetylene.

Due to limited observations of OVOCs and the differences in measured VOC species, the average concentrations of 57 PAMS VOCs from some studies were compared to better understand the VOC pollution levels in different Chinese cities (Table 1). Compared with other Chinese cities, the average VOCs concentration in this study was higher than that in Wuhan [44], but lower than those in Jinzhong [45], Xi’an [46], Luoyang [47], Zhengzhou [42] and Beijing [48]. Sampling during peak photochemical activity (12:00–15:00) may have led to lower observed VOC concentrations due to photochemical losses [49].

### 3.2. Ozone Formation Potential

**Figure 4:**
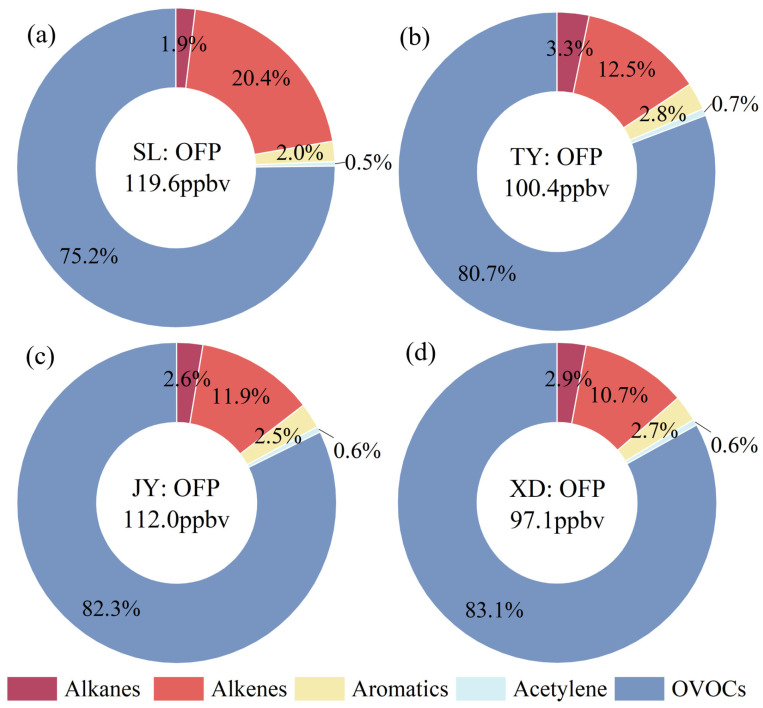
Contributions of VOC groups to OFP at the (**a**) SL, (**b**) TY, (**c**) JY and (**d**) XD.

**Figure 5:**
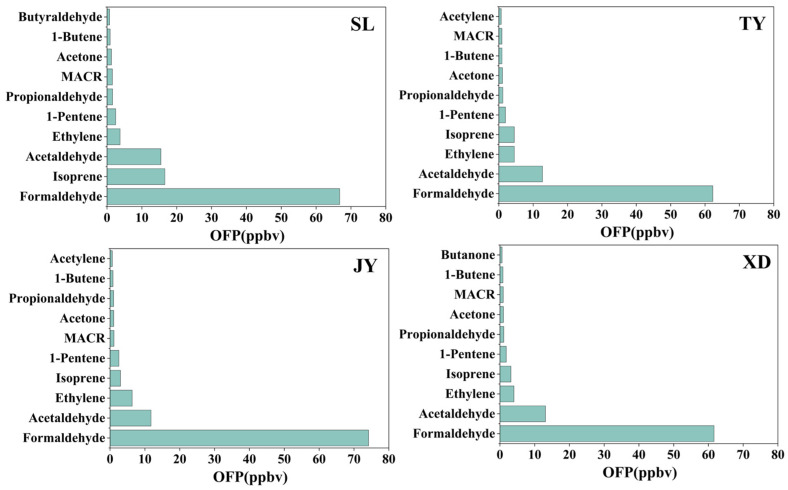
OFP of the top 10 VOC species at SL, TY, JY and XD.

Ozone formation potential (OFP) is a theoretical tool to estimate the maximum O_3_ production of each VOC species under optimum conditions. By comparing the OFP values of different VOCs, the species that contribute most to local ozone formation can be identified. Detailed information about the OFP of five VOC classes to TVOCs at four sites is shown in Figure 4. The OFP values of TVOCs at SL, TY, JY and XD sites were 119.6 ± 47.7 ppbv, 100.4 ± 34.2 ppbv, 112.0 ± 58.2 ppbv, 97.1 ± 34.1 ppbv, respectively. The contributions of five VOC groups to OFP at four sites ranked as follows: OVOCs (75.2~83.1%) > alkenes (10.7~20.4%) > alkanes (1.9~3.3%) ≈ aromatics (2.0~2.8%) > acetylene (0.5~0.7%). For individual species, the top 10 contributors to OFP were presented in Figure 5 and Table S5, accounting for 90.2~92.9% of the total OFP from TVOCs across the four sites. Among them, formaldehyde, acetaldehyde, isoprene and ethylene were the greatest contributors to OFP in Taiyuan, reaching 83.6~85.7% of the total OFP at the four sites. These VOC species are key targets for mitigating O_3_ pollution in Taiyuan.

OVOCs were the biggest contributor to OFP, accounting for 75.2%, 80.7%, 82.3% and 83.1% at SL, TY, JY and XD, respectively, which was similar to those in Beijing [19], Zibo [20] and Baoji [21]. The finding that OVOCs contributed over 75% to OFP across all sites underscores the critical role of oxygenated species in O_3_ formation in Taiyuan. The contribution of OVOCs in Taiyuan is higher than those reported in Beijing (45.2%) [19] and Zibo (46.0–55.9%) [20], likely due to the combined influence of strong local photochemistry and regional transport of aged air masses in the Taiyuan Basin [18]. Compared to our previous study at JY, which measured only PAMS VOCs and found that alkenes (71.9%) dominated OFP [3], the inclusion of OVOCs in this study reveals a substantially higher contribution from secondary sources. This comparison emphasizes the importance of simultaneous PAMS VOCs and OVOCs measurements for accurate OFP assessment. Regarding the OFP of individual species, formaldehyde was not only the dominant OVOC species, but also the biggest contributor for all studied VOC species at four sites in this study. Most results showed that secondary formation (40–70%) is the main source of formaldehyde [50,51]. Therefore, reducing reactive VOC precursors, such as alkenes and aromatics, may be an effective strategy to lower formaldehyde levels and subsequently mitigate O_3_ formation. As shown in Table S5, the average OFP value of formaldehyde at SL, TY, JY and XD varied from 61.6 to 74.2 ppbv, accounting for 55.8~66.2% of the total OFP. The second-largest contributor to OFP among OVOCs was acetaldehyde at TY, JY, and XD, accounting for 10.5~13.5% of total OFP. Among the VOC groups, alkenes constituted the second largest contributor to total OFP, representing 20.4%, 12.5%, 11.9% and 10.7% at SL, TY, JY and XD, respectively. The highest contribution of alkenes to OFP was observed at SL, which was mainly attributed to the isoprene emissions from biological sources in the vicinity of this site. Isoprene was the second-largest contributor at SL with a proportion of 13.9%. By contrast, as shown in Figure 5 and Table S5, the OFP of ethylene at TY, JY and XD was higher than that of isoprene. In our previous study at JY [3], a total of 58 VOCs (29 alkanes, 11 alkenes, 17 aromatics, and 1 acetylene) were measured. The OFP analysis results in summer indicated that alkenes (71.9%) and ethylene were the most significant contributors and the most abundant VOCs specie, respectively. For alkanes, aromatics and acetylene, due to their relatively low MIR values, the contributions of these three VOC groups to the total OFP were within the range of 4.4~6.8%.

### 3.3. Source Apportionment of VOCs

**Figure 6:**
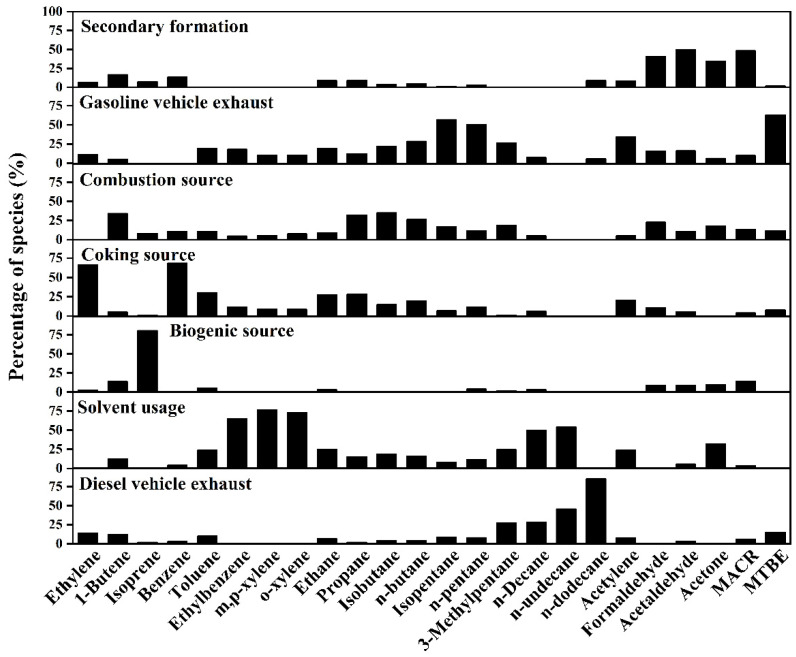
Factor profiles of secondary source, gasoline vehicle exhaust, combustion source, coking source, biogenic source, solvent use and diesel vehicle exhaust resolved by PMF.

**Figure 7:**
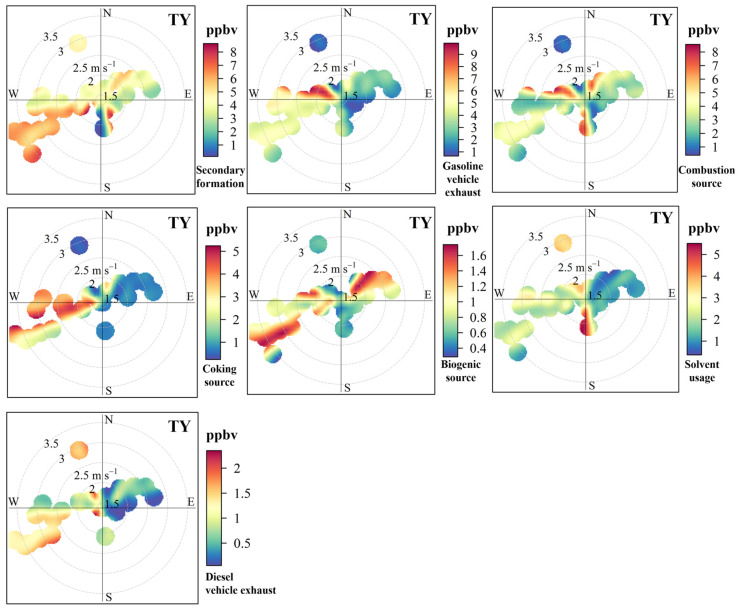
Wind plots for seven pollution source concentrations at TY.

**Figure 8:**
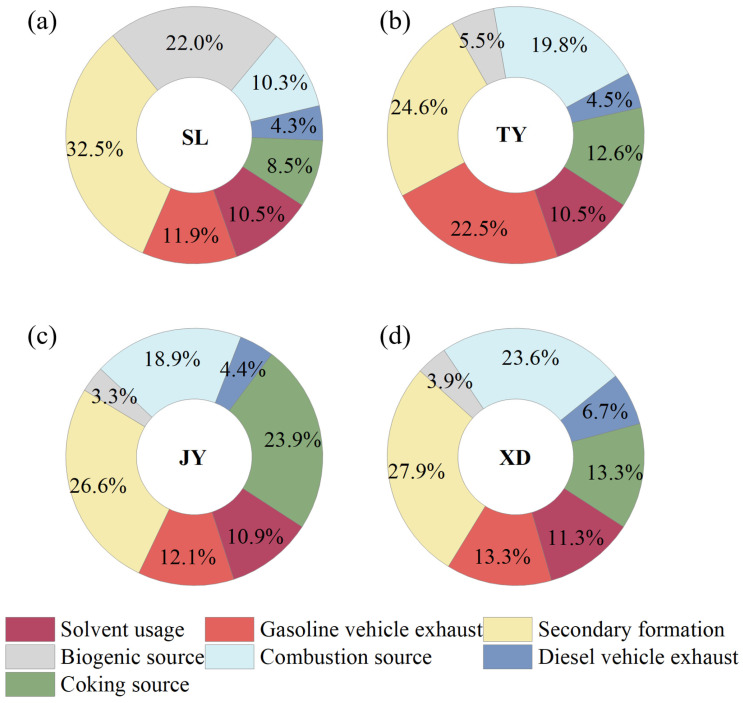
Contribution of each emission source to VOCs at (**a**) SL, (**b**) TY, (**c**) JY and (**d**) XD.

**Figure 9:**
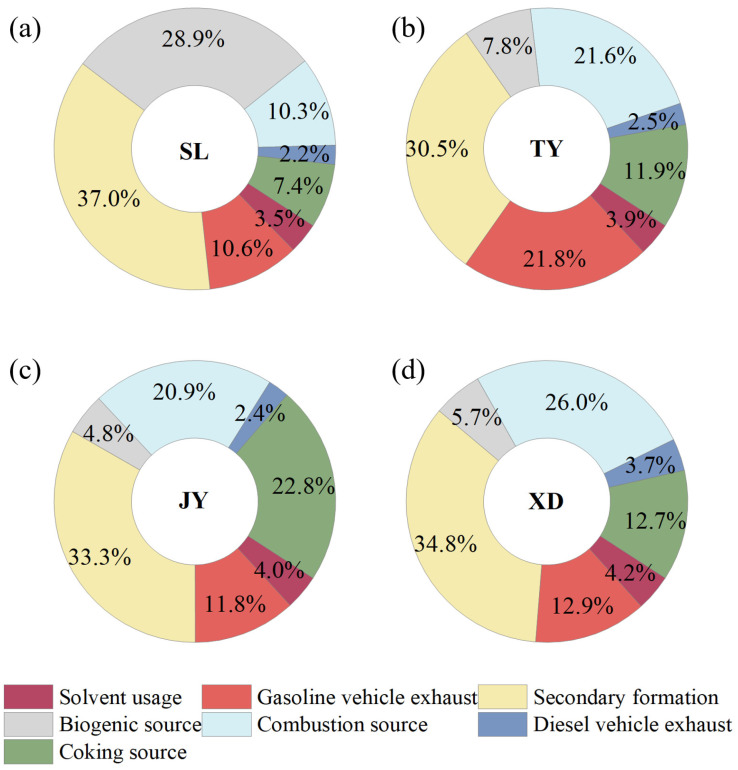
Contribution of each emission source to OFP at (**a**) SL, (**b**) TY, (**c**) JY and (**d**) XD.

The factor profiles of VOC sources resolved by the PMF model are shown in Figure 6. Seven sources were identified for VOCs in Taiyuan, including secondary formation, gasoline vehicle exhaust, combustion source, coking source, biogenic source, solvent usage, and diesel vehicle exhaust. The wind rose plots of seven VOCs sources at TY (Figure 7), SL (Figure S4), JY (Figure S5) and XD (Figure S6) were used to support the reasonability of source assignments. To match the 3 h integrated VOCs samples with hourly meteorological data, wind direction was calculated as the vector average, while wind speed was calculated as the arithmetic average over the corresponding sampling period. The wind plots were obtained using the openair package in R. Specifically, we used the polarPlot functions with pollutant concentration as a conditional variable. The source contributions of VOC concentrations at four sites are displayed in Figure 8. Furthermore, the contributions of different VOC sources to the OFP were calculated using the source profiles from PMF and corresponding MIR coefficients, and the results are shown in Figure 9.

Factor 1 was characterized by a higher proportion of formaldehyde (40.9%), acetaldehyde (49.4%), acetone (34.1%) and MACR (48.4%). Besides a fraction from primary anthropogenic emissions, OVOCs in the atmosphere mainly originate from the secondary formation through photochemical reactions of reactive VOCs (e.g., alkene) in summer [24,52,53,54]. MACR is the oxidation product of isoprene [53,55]. As seen from Figure 7 at TY, except for some higher values caused by local photochemical reactions at lower WS (<2 m/s), the wind rose plots showed that higher concentrations were associated with higher WS (>2 m/s). This was because reactive VOCs from the aged air masses could undergo photochemical reactions during the transport process [27]. Therefore, this factor was identified as secondary formation. The spatial distribution of contribution from secondary formation to VOC concentrations was as follows: SL (32.5%) > XD (27.9%) > JY (26.6%) > TY (24.6%), and it was the largest source of VOCs across the four sites. The corresponding OFP contribution showed a similar pattern: SL (37.0%) > XD (34.8%) > JY (33.3%) > TY (30.5%). The highest contributions of secondary sources in terms of concentration and OFP were observed at SL, which could likely be attributed to the significant influence of secondary formation from biogenic alkenes (e.g., isoprene) at this site.

Factor 2 showed higher loadings of ethane (19.4%), isopentane (56.9%), n-pentane (50.7%), 3-methylpentane (26.2%), acetylene (34.1%), and MTBE (62.8%). 3-Methylpentane, n-pentane, and isopentane are associated with gasoline vehicle emissions [24,42]. MTBE is also a well-known tracer for gasoline vehicles [2], showing the highest contribution in this factor. Thus, factor 2 could be regarded as gasoline vehicle exhaust. The spatial distribution of its contribution to TVOCs is distributed as TY (22.5%) > XD (13.3%) > JY (12.1%) > SL (11.9%). The OFP contribution followed a similar order: TY (21.8%) > XD (12.9%) > JY (11.8%) > SL (10.6%). The TY site is located in the central urban area in Taiyuan, and is about 1 km away from two major roads (Binhe West Road and Binhe East Road) with heavy traffic flows. The results from wind plots in Figure 7 further supported the identification of this source as gasoline vehicle exhaust.

Factor 3 was distinguished by higher contributions from propane (32.4%), isobutane (35.1%), and n-butane (26.4%). These VOC species are linked to coal combustion and natural gas/liquefied petroleum gas (NG/LPG) usage [25,42]. Therefore, this factor was identified as a combustion source. The spatial distribution of concentration contribution from combustion sources was as follows: XD (23.6%) > TY (19.8%) > JY (18.9%) > SL (10.3%). The OFP contribution followed a similar order: XD (26.0%) > TY (21.6%) > JY (20.9%) > SL (10.3%). XD exhibited the highest contribution for both concentration and OFP, which may be related to NG/LPG usage in surrounding catering services, as well as coal-combustion-related activities situated to the southwest of this sampling site (Figure 1). The wind rose plot of the combustion source at TY (Figure 7) could also support the identification of factor 3.

Factor 4 was characterized by higher loadings of ethylene (66.2%), benzene (68.5%), ethane (27.6%) and acetylene (20.5%). According to our previous studies, these species are primarily emitted from various stages of the coking process, particularly ethylene and benzene [2,3]. Hence, Factor 4 was identified as a coking source. The wind rose plots results of the coke source at four sites were consistent with the distribution of coking plants (Figure 1). For instance, a higher coking source contribution was observed in the north of the TY site under low WS, which can be ascribed to the influence of coking processes from the nearby large iron and steel plant (Figure 7). Additionally, due to the effect of intensive coking plants, most higher values of coking source mainly originated from the southwest of TY at a higher WS (>2 m/s). The spatial distribution of its concentration contribution ranked as follows: JY (23.9%) > XD (13.3%) > TY (12.6%) > SL (8.5%). The corresponding OFP contribution was as follows: JY (22.8%) > XD (12.7%) > TY (11.9%) > SL (7.4%). The highest contributions for both concentration and OFP occurred at the JY site, which could be attributed to the VOCs emission from coking plants located in the south of the site and Jinzhong [45].

Factor 5 was dominated by isoprene (80.2%), a compound traditionally regarded as a tracer for biogenic emissions [2,3,24,56]. However, a growing body of the literature has documented anthropogenic sources of isoprene, including vehicular exhaust [57], biomass burning [58,59], and industrial activities [60]. In our study, isoprene exhibited weak correlations with typical anthropogenic tracers (MTBE, R^2^ = 0.05; acetylene, R^2^ = 0.00; benzene, R^2^ = 0.01). Furthermore, the contribution of Factor 5 showed a clear spatial gradient that closely aligned with vegetation cover (Figure 1b). Taken together, we identify Factor 5 as a primarily biogenic source, while acknowledging that a minor contribution from anthropogenic isoprene cannot be entirely excluded. The spatial distribution of concentration contribution was SL (22.0%) > TY (5.5%) > XD (3.9%) > JY (3.3%). Its OFP contribution was as follows: SL (28.9%) > TY (7.8%) > XD (5.7%) > JY (4.8%). SL showed the highest contributions for both concentration and OFP, indicating the significant influence of vegetation emission from the nearby mountains. The wind rose plots of this factor at the four sites were consistent with the distribution of vegetation around the sites during the sampling period (Figure 1). As presented in Figure 7, elevated biogenic emission at TY was associated with low WS (<2 m/s) from the west and higher WS (>2 m/s) from the northeast and southwest. This pattern aligned with the local vegetation distribution: the Fenhe Wetland Park, a known biogenic source, is located about 1 km west of TY, while emissions at higher WS likely originated from the vegetated mountain areas to the northeast and southwest of Taiyuan.

Factor 6 was enriched with toluene (23.7%), ethylbenzene (64.9%), m/p-xylene (76.1%), o-xylene (72.9%), n-decane (49.7%), n-undecane (54.0%), and acetone (32.2%). Aromatics (e.g., toluene, ethylbenzene) and acetone are widely associated with solvent usage in industries such as painting and printing [2,3], while n-decane and n-undecane are linked to asphalt volatilization from road surfaces in summer [21]. Therefore, this factor was identified as solvent usage. For example, a higher solvent usage contribution was observed south of the TY site, which was closely related to nearby construction activities, such as painting and road paving (Figure 7). The spatial distribution of its concentration contribution was XD (11.3%) > JY (10.9%) > TY (10.5%) = SL (10.5%). The corresponding OFP contribution was as follows: XD (4.2%) > JY (4.0%) > TY (3.9%) > SL (3.5%). The contributions of solvent usage to concentration and OFP at each site were approximately 10% and 4%, respectively.

Factor 7 was characterized by high loadings of 3-methylpentane (27.4%), n-decane (28.4%), n-undecane (45.5%), and n-dodecane (85.4%). n-Undecane is a known diesel fuel additive, and long-chain alkanes are primarily emitted from diesel vehicle exhaust [53]. Thus, Factor 7 was identified as diesel vehicle exhaust. The spatial distribution of its concentration contribution was XD (6.7%) > TY (4.5%) > JY (4.4%) > SL (4.3%). Its OFP contribution was as follows: XD (3.7%) > TY (2.5%) > JY (2.4%) > SL (2.2%). Wind rose plots showed higher concentration distribution of this factor was consistent with the distribution of main trunk traffic roads near the sampling sites. As shown in Figure 7, elevated contributions from diesel vehicle exhaust at the TY site were predominantly observed under low WS (<2 m/s), which could be directly linked to the nearby roads (Binhe West and East roads) to the west of TY. XD showed the highest contributions to both concentration and OFP, likely due to the influence of the freeway entrance around this sampling site (Figure S1).

In conclusion, secondary formation was the largest contributor to both VOC concentrations (24.6–32.5%) and OFP (30.5–37.0%) at all sites, highlighting the regional nature of secondary pollution in Taiyuan. This finding aligns with studies in Beijing (27.8%) [19] and Chongqing (27.7%) [39], suggesting that reducing reactive VOC precursors is essential for O_3_ abatement in many Chinese cities. Notably, the second-largest source exhibited systematic spatial heterogeneity, providing critical insights for targeted control strategies. At TY, gasoline vehicle exhaust was the second-largest contributor (22.5% to concentration, 21.8% to OFP), reflecting heavy traffic flows. Prioritizing vehicle emission controls, such as tightening fuel standards, promoting new energy vehicles, and implementing traffic restrictions during high-O_3_ episodes, is recommended for central Taiyuan. At XD, combustion sources (likely NG/LPG usage) ranked second (23.6% to concentration, 26.0% to OFP), suggesting that reducing emissions from catering services could effectively lower VOC levels. At JY, coking sources were the second-largest contributor (23.9% to concentration, 22.8% to OFP). Coking emissions remain a key control target, requiring enhanced fugitive capture, continuous monitoring, and regional cooperation with the Qingxu industrial park. A previous study conducted at the JY site in the summer of 2021, which measured only PAMS VOCs, reported that coking sources were the primary contributor to VOCs (36.1%) based on PMF analysis. This suggests that the lack of OVOCs may lead to an overestimation of primary sources and an underestimation of photochemical processing in source apportionment studies in this region. At SL, biogenic sources ranked second (22.0% to concentration, 28.9% to OFP). While direct biogenic control is impractical, monitoring regional transport from industrial areas is warranted. Therefore, zone-specific measures should be complemented by regional precursor reductions across the Taiyuan Basin to abate O_3_ pollution effectively.

## 4. Conclusions

### 4.1. General Findings

In this study, 57 PAMS and 14 OVOC concentrations were measured simultaneously at three urban sites and a rural site in Taiyuan, and spatial variations, main components, OFP, and their main sources were analyzed. The spatial distribution of TVOC concentrations was characterized by higher levels in the northern (SL) and southern regions (JY) and lower levels in the central urban region (TY and XD). OVOCs were the most abundant components of VOCs, accounting for more than 60% of TVOCs. Formaldehyde, acetone, and acetaldehyde were the three most abundant VOC species at the four sites. OVOCs were the predominant contributors to OFP at the four sites, accounting for more than 75%. Formaldehyde was identified as the most reactive VOC species at the four sites. Seven major sources of VOCs were identified by the PMF model, including secondary formation, combustion source, gasoline vehicle exhaust, coking source, biogenic source, solvent usage and diesel vehicle exhaust. Secondary formation was the largest source of VOCs at SL (32.5%), TY (24.6%), JY (26.6%) and XD (27.9%). Biogenic source, gasoline vehicle exhaust, coking source and combustion source were the second-largest VOCs contributors at SL (22.0%), TY (22.5%), JY (23.9%) and XD (23.6%), respectively. The source apportionment results for OFP were consistent with those for VOCs concentrations. This study highlighted the influence of OVOCs on O_3_ formation and clarified the spatial discrepancies of characteristics and sources of VOCs in different areas at the city level. Given the critical role of secondary formation source in the formation of O_3_, detailed studies on the mechanism and reapportionment of secondary formation source from OVOCs should be further conducted in future research.

### 4.2. Limitations and Future Research

This study has several limitations that should be acknowledged. First, the sampling strategy, which collected samples once every six days during afternoon hours only (12:00–15:00), captures peak photochemical conditions but does not capture diurnal or weekly variations in VOCs. Future work should employ continuous online monitoring to more accurately capture source variability and inform O_3_ control strategies. Second, our PMF analysis pooled samples from all four sites to achieve sufficient statistical power, which assumes consistent source profiles across sites—an assumption partially validated by wind rose analysis but warranting further investigation with larger datasets. Third, given the critical role of secondary formation in O_3_ production, more detailed studies on the formation mechanisms and reapportionment of secondary sources from OVOCs should be conducted in future research.

## Figures and Tables

**Table 1:** Comparisons of 57 PAMS concentrations (ppbv) observed in Taiyuan and other cities.

Locations		Sampling Time	VOCs	Reference
Taiyuan	SL	June–August 2022 June–August 2023	7.2 ± 3.7	This study
	TY		7.3 ± 3.1	
	JY		7.8 ± 4.8	
	XD		6.7 ± 3.4	
Jinzhong		April–September 2021	12.9	[45]
Beijing		1 August–29 August 2019	11.1	[48]
Xi’an		16 July–28 July 2018	19.0	[46]
Luoyang		6 July–10 August 2019	20.9 ± 10.3	[47]
Zhengzhou		June–August 2017	24.7	[42]
Wuhan		August 2019	8.8	[44]

## Data Availability

The original contributions presented in this study are included in the article. Further inquiries can be directed to the corresponding authors.

## Supplementary Materials

The following supporting information can be downloaded at: https://www.mdpi.com/article/10.3390/toxics14030220/s1, Figure S1: Detailed information on the surroundings of the sampling sites; Figure S2: Monthly and diurnal variations in O_3_ in Taiyuan in 2022 and 2023; Figure S3: Wind rose diagram during the sampling periods at SL, TY, JY and XD; Figure S4: Wind plots for seven pollution source concentrations at SL; Figure S5: Wind plots for seven pollution source concentrations at JY; Figure S6: Wind plots for seven pollution source concentrations at XD; Table S1: List of method detection limits in 2022 and 2023 (ppbv); Table S2: The correlation coefficients (R^2^) between observed and modeled values for each VOC species by PMF; Table S3: Summary of PMF error estimation diagnostics from BS; Table S4: Concentration levels of VOCs in Taiyuan (ppbv); Table S5: The top 10 VOCs ranked according to calculated OFP.

## References

[1] Liu Y., Geng G., Cheng J., Liu Y., Xiao Q., Liu L., Shi Q., Tong D., He K., Zhang Q. (2023). Drivers of Increasing Ozone during the Two Phases of Clean Air Actions in China 2013–2020. Environ. Sci. Technol..

[2] Ren X., Wen Y., He Q., Cui Y., Gao X., Li F., Wang Y., Guo L., Li H., Wang X. (2021). Higher Contribution of Coking Sources to Ozone Formation Potential from Volatile Organic Compounds in Summer in Taiyuan, China. Atmos. Pollut. Res..

[3] Wang Y., Cui Y., He Q., Fan J., Li Y., Liu K., Guo L., Wang X. (2023). Significant Impact of VOCs Emission from Coking and Coal/Biomass Combustion on O_3_ and SOA Formation in Taiyuan, China. Atmos. Pollut. Res..

[4] Juráň S., Karl T., Ofori-Amanfo K.K., Šigut L., Zavadilová I., Grace J., Urban O. (2025). Drought shifts ozone deposition pathways in spruce forest from stomatal to non-stomatal flux. Environ. Pollut..

[5] Feng Z., De Marco A., Anav A., Gualtieri M., Sicard P., Tian H., Fornasier F., Tao F., Guo A., Paoletti E. (2019). Economic losses due to ozone impacts on human health, forest productivity and crop yield across China. Environ. Int..

[6] Karlsson P.E., Klingberg J., Engardt M., Andersson C., Langner J., Karlsson G.P., Pleijel H. (2017). Past, present and future concentrations of ground-level ozone and potential impacts on ecosystems and human health in northern Europe. Sci. Total Environ..

[7] Wang W., Parrish D.D., Wang S., Bao F., Ni R., Li X., Yang S., Wang H., Cheng Y., Su H. (2022). Long-Term Trend of Ozone Pollution in China during 2014–2020: Distinct Seasonal and Spatial Characteristics and Ozone Sensitivity. Atmos. Chem. Phys..

[8] Wang F., Du W., Lv S., Ding Z., Wang G. (2021). Spatial and Temporal Distributions and Sources of Anthropogenic NMVOCs in the Atmosphere of China: A Review. Adv. Atmos. Sci..

[9] Liu B., Liang D., Yang J., Dai Q., Bi X., Feng Y., Yuan J., Xiao Z., Zhang Y., Xu H. (2016). Characterization and Source Apportionment of Volatile Organic Compounds Based on 1-Year of Observational Data in Tianjin, China. Environ. Pollut..

[10] Liu Y., Yin S., Zhang S., Ma W., Zhang X., Qiu P., Li C., Wang G., Hou D., Zhang X. (2024). Drivers and Impacts of Decreasing Concentrations of Atmospheric Volatile Organic Compounds (VOCs) in Beijing during 2016–2020. Sci. Total Environ..

[11] Yang S., Li X., Song M., Liu Y., Yu X., Chen S., Lu S., Wang W., Yang Y., Zeng L. (2021). Characteristics and Sources of Volatile Organic Compounds during Pollution Episodes and Clean Periods in the Beijing-Tianjin-Hebei Region. Sci. Total Environ..

[12] Shao M., Zhang Y., Zeng L., Tang X., Zhang J., Zhong L., Wang B. (2009). Ground-Level Ozone in the Pearl River Delta and the Roles of VOC and NOx in Its Production. J. Environ. Manag..

[13] An J., Zhu B., Wang H., Li Y., Lin X., Yang H. (2014). Characteristics and Source Apportionment of VOCs Measured in an Industrial Area of Nanjing, Yangtze River Delta, China. Atmos. Environ..

[14] Xiao Z., Yang X., Gu H., Hu J., Zhang T., Chen J., Pan X., Xiu G., Zhang W., Lin M. (2024). Characterization and Sources of Volatile Organic Compounds (VOCs) during 2022 Summer Ozone Pollution Control in Shanghai, China. Atmos. Environ..

[15] Wang Y., Ren X., Ji D., Zhang J., Sun J., Wu F. (2012). Characterization of Volatile Organic Compounds in the Urban Area of Beijing from 2000 to 2007. J. Environ. Sci..

[16] Sun W., Shao M., Granier C., Liu Y., Ye C.S., Zheng J.Y. (2018). Long-Term Trends of Anthropogenic SO_2_, NO_X_, CO, and NMVOCs Emissions in China. Earth’s Future.

[17] Jia C., Mao X., Huang T., Liang X., Wang Y., Shen Y., Jiang W., Wang H., Bai Z., Ma M. (2016). Non-Methane Hydrocarbons (NMHCs) and Their Contribution to Ozone Formation Potential in a Petrochemical Industrialized City, Northwest China. Atmos. Res..

[18] Liu Z., Cui Y., He Q., Guo L., Gao X., Feng Y., Wang Y., Wang X. (2021). Seasonal Variations of Carbonyls and Their Contributions to the Ozone Formation in Urban Atmosphere of Taiyuan, China. Atmosphere.

[19] Liu C., Xin Y., Zhang C., Liu J., Liu P., He X., Mu Y. (2023). Ambient Volatile Organic Compounds in Urban and Industrial Regions in Beijing: Characteristics, Source Apportionment, Secondary Transformation and Health Risk Assessment. Sci. Total Environ..

[20] Dai J., Zhang K., Feng Y., Yi X., Li R., Xue J., Li Q., Shi L., Liao J., Yi Y. (2025). Significant Influence of Oxygenated Volatile Organic Compounds on Atmospheric Chemistry: A Case Study in a Typical Industrial City in China. Atmos. Chem. Phys..

[21] Pang X., Chen L., Gao B., Wang S., Zhao W., Liu M., Lu H., Liang X. (2023). Characteristics and Health Risk Assessment of Volatile Organic Compounds in Different Functional Zones in Baoji in Summer. Environ. Sci..

[22] Wang C., Huang X., Han Y., Zhu B., He L. (2017). Sources and Potential Photochemical Roles of Formaldehyde in an Urban Atmosphere in South China. J. Geophys. Res. Atmos..

[23] Ho K.F., Ho S.S.H., Dai W.T., Cao J.J., Huang R.-J., Tian L., Deng W.J. (2014). Seasonal Variations of Monocarbonyl and Dicarbonyl in Urban and Sub-Urban Sites of Xi’an, China. Environ. Monit. Assess..

[24] Hua J., Cui Y., Guo L., Li H., Fan J., Li Y., Wang Y., Liu K., He Q., Wang X. (2023). Spatial Characterization of HCHO and Reapportionment of Its Secondary Sources Considering Photochemical Loss in Taiyuan, China. Sci. Total Environ..

[25] Han Y., Wang T., Li R., Fu H., Duan Y., Gao S., Zhang L., Chen J. (2023). Measurement Report: Volatile Organic Compound Characteristics of the Different Land-Use Types in Shanghai: Spatiotemporal Variation, Source Apportionment and Impact on Secondary Formations of Ozone and Aerosol. Atmos. Chem. Phys..

[26] Wei Y., Jing X., Chen Y., Sun W., Zhang Y., Zhu R. (2024). Spatial–Temporal Characteristics, Source Apportionment, and Health Risks of Atmospheric Volatile Organic Compounds in China: A Comprehensive Review. Toxics.

[27] Song M., Li X., Yang S., Yu X., Zhou S., Yang Y., Chen S., Dong H., Liao K., Chen Q. (2021). Spatiotemporal Variation, Sources, and Secondary Transformation Potential of Volatile Organic Compounds in Xi’an, China. Atmos. Chem. Phys..

[28] Ren J., Guo F., Xie S. (2022). Diagnosing Ozone–NOx–VOC Sensitivity and Revealing Causes of Ozone Increases in China Based on 2013–2021 Satellite Retrievals. Atmos. Chem. Phys..

[29] Zhu C., Gai Y., Liu Z., Sun L., Fu S., Liu K., Yang L., Pan G., Wang B., Wang C. (2024). Long-Term Changes of Surface Ozone and Ozone Sensitivity over the North China Plain Based on 2015–2021 Satellite Retrievals. Air Qual. Atmos. Health.

[30] (2017). Technical Specification on Manual Methods for Ambient Air Quality Monitoring.

[31] (2012). Ambient Air Quality Standards.

[32] Huang X., Gao L. (2025). Air Pollution in Taiyuan City During 2022 to 2024: Status and Influence of Meteorological Factors. Atmosphere.

[33] Zou Y., Charlesworth E., Wang N., Flores R.M., Liu Q.Q., Li F., Deng T., Deng X.J. (2021). Characterization and Ozone Formation Potential (OFP) of Non-Methane Hydrocarbons under the Condition of Chemical Loss in Guangzhou, China. Atmos. Environ..

[34] Carter W.P.L. (2010). Development of the SAPRC-07 Chemical Mechanism. Atmos. Environ..

[35] Song Y., Shao M., Liu Y., Lu S., Kuster W., Goldan P., Xie S. (2007). Source Apportionment of Ambient Volatile Organic Compounds in Beijing. Environ. Sci. Technol..

[36] Zhang B., Zhang J., Jiang N., Wang Q., Wang Y., Wang C., Shen X. (2025). Evaluation and Analysis of the Pollution Characteristics of Oxygenated Volatile Organic Compounds in Beijing and Their Impact on Ozone Formation. Res. Environ. Sci..

[37] Zhang H., Sui H., Wang Z., Zhang S., Du M., Ge X., Wang M., Tao W., Xu H., Gu D. (2024). Study on Characteristics and Sources of Volatile Organic Compounds in Urban Jinan, China. China Environ. Sci..

[38] Qian X., Shen H., Chen Z. (2019). Characterizing Summer and Winter Carbonyl Compounds in Beijing Atmosphere. Atmos. Environ..

[39] Li L., Li Z., Zhang D., Fang W.K., Xu Q., Duan L., Lu P., Wang F., Zhang W., Zhai C. (2021). Pollution characteristics and source apportionment of atmospheric VOCs during ozone pollution period in the main urban area of Chongqing. Environ. Sci..

[40] Liu G., Ma X., Li W., Chen J., Ji Y., An T. (2024). Pollution characteristics, source appointment and environmental effect of oxygenated volatile organic compounds in Guangdong-Hong Kong-Macao Greater Bay Area: Implication for air quality management. Sci. Total Environ..

[41] Cui J., Sun M., Wang L., Guo J., Xie G., Zhang J., Zhang R. (2021). Gas-Particle Partitioning of Carbonyls and Its Influencing Factors in the Urban Atmosphere of Zhengzhou, China. Sci. Total Environ..

[42] Li B., Ho S.S.H., Gong S., Ni J., Li H., Han L., Yang Y., Qi Y., Zhao D. (2019). Characterization of VOCs and Their Related Atmospheric Processes in a Central Chinese City during Severe Ozone Pollution Periods. Atmos. Chem. Phys..

[43] Xiao L., Wang S., Zhou Y., Chai W., Du L., Tang G., Li J. (2021). The Characteristics and Source Apportionments of VOCs at Typical Background Sites during Summer in China. China Environ. Sci..

[44] Wang R., Wang L., Xue M., Chen N., Zhang L., Ling Z., Li T., Tao M., Wang Y. (2023). New Insight into Formation Mechanism, Source and Control Strategy of Severe O_3_ Pollution: The Case from Photochemical Simulation in the Wuhan Metropolitan Area, Central China. Atmos. Res..

[45] Wang Z., Long T., Cui Y., He Q., Wang J., Gao S., Wang X. (2024). Annual Variations in Characteristics and Sources Analysis of VOCs during the Ozone Season in the Taiyuan Basin, China, from 2020 to 2022. Air Qual. Atmos. Health.

[46] Sun J., Shen Z., Zhang Y., Zhang Z., Zhang Q., Zhang T., Niu X., Huang Y., Cui L., Xu H. (2019). Urban VOC Profiles, Possible Sources, and Its Role in Ozone Formation for a Summer Campaign over Xi’an, China. Environ. Sci. Pollut. Res..

[47] Sun J., Shen Z., Wang R., Li G., Zhang Y., Zhang B., He K., Tang Z., Xu H., Qu L. (2021). A Comprehensive Study on Ozone Pollution in a Megacity in North China Plain during Summertime: Observations, Source Attributions and Ozone Sensitivity. Environ. Int..

[48] Li Y., Tang J., Dong M., Zhou F., Qi J., Gao R., Hu Y., Zhang X., Wang Y., Li H. (2025). Comparative Analysis of Pollution Characteristics and Sources of Ambient VOCs Across Typical Functional Districts of Beijing. Res. Environ. Sci..

[49] Zhan J., Feng Z., Liu P., He X., He Z., Chen T., Wang Y., He H., Mu Y., Liu Y. (2021). Ozone and SOA Formation Potential Based on Photochemical Loss of VOCs during the Beijing Summer. Environ. Pollut..

[50] Huang H., Yang C., Wang Z., Lian S., Li X., Liu Y., Cheng H. (2024). The chemical characteristics and sources of formaldehyde on O_3_ and non-O_3_ polluted days in Wuhan, central China. Atmos. Environ..

[51] Bao J., Li H., Wu Z., Zhang X., Zhang H., Li Y., Qian J., Chen J., Deng L. (2022). Atmospheric carbonyls in a heavy ozone pollution episode at a metropolis in Southwest China: Characteristics, health risk assessment, sources analysis. J. Environ. Sci..

[52] Zhang H., Zhang C., Liu S., Yin S., Zhang S., Zhu H., Yan F., Yang H., Ru X., Liu X. (2025). Insights into the Source Characterization, Risk Assessment and Ozone Formation Sensitivity of Ambient VOCs at an Urban Site in the Fenwei Plain, China. J. Hazard. Mater..

[53] Li Y., Li H., Zhang X., Ji Y., Gao R., Wu Z., Yin M., Nie L., Wei W., Li G. (2023). Characteristics, Sources and Health Risk Assessment of Atmospheric Carbonyls during Multiple Ozone Pollution Episodes in Urban Beijing: Insights into Control Strategies. Sci. Total Environ..

[54] Nogueira T., Dominutti P.A., Fornaro A., Andrade M.D.F. (2017). Seasonal trends of formaldehyde and acetaldehyde in the megacity of São Paulo. Atmosphere.

[55] Gu C., Wang S., Zhu J., Wu S., Duan Y., Gao S., Zhou B. (2022). Investigation on the Urban Ambient Isoprene and Its Oxidation Processes. Atmos. Environ..

[56] Ahmed M., Ahmad M., Rappenglueck B. (2025). Twenty years (2004–2023) observation of non-methane hydrocarbons in a subtropical coastal environment–Indications of increased isoprene emissions. Atmos. Environ..

[57] Khan M., Schlich B., Jenkin M., Shallcross B., Moseley K., Walker C., Morris R., Derwent R., Percival C., Shallcross D. (2018). A two-decade anthropogenic and biogenic isoprene emissions study in a London urban background and a London urban traffic site. Atmosphere.

[58] Zhang Y., Men Y., Guo H., Shen G., Gao Y., Xiong R., Tao S., Wang X. (2025). Combustion-related isoprene contributes substantially to the formation of wintertime secondary organic aerosols. Natl. Sci. Rev..

[59] Andreae M. (2019). Emission of trace gases and aerosols from biomass burning-an updated assessment. Atmos. Chem. Phys..

[60] Cai C., Geng F., Tie X., Yu Q., Peng L., Zhou G. (2010). Characteristics of ambient volatile organic compounds (VOCs) measured in Shanghai, China. Sensors.

