# Supplementary material for: Spatial Characteristics, Sources of Volatile Organic Compounds and Effects on O3 Formation in Summer in Taiyuan, China
Source: Toxics. 2026 Mar 4;14(3):220. doi: 10.3390/toxics14030220 (PMC13030084; doi:10.3390/toxics14030220)
Supplement: Supplementary file 1 [file toxics-14-00220-s001.zip › toxics-4116427-supplementary.pdf]

Supplementary material

Spatial characteristics, sources of volatile organic compounds and effects on O_3 formation in summer in Taiyuan, China

Lili Guo [1,†], Tianyu Gao [2,†], Bingxi Wang [2], Yang Cui [2,*], Qiusheng He [1,2,*], Zhentao Wang [2], Xiaojing Hu [2] and Xinming Wang [3]

[1] School of Materials Science and Engineering, Taiyuan University of Science and Technology, Taiyuan 030024, China

[2] School of Environment and Resources, Shanxi Key Laboratory of Coordinated Management and Control for Environmental Quality, Taiyuan University of Science and Technology, Taiyuan 030024, China

3 State Key Laboratory of Organic Geochemistry, Guangzhou Institute of Geochemistry, Chinese Academy of Sciences, Guangzhou 510640, China

**Corresponding author: 2020052@tyust.edu.cn; heqs@tyust.edu.cn*

† These authors contributed equally to this work[M

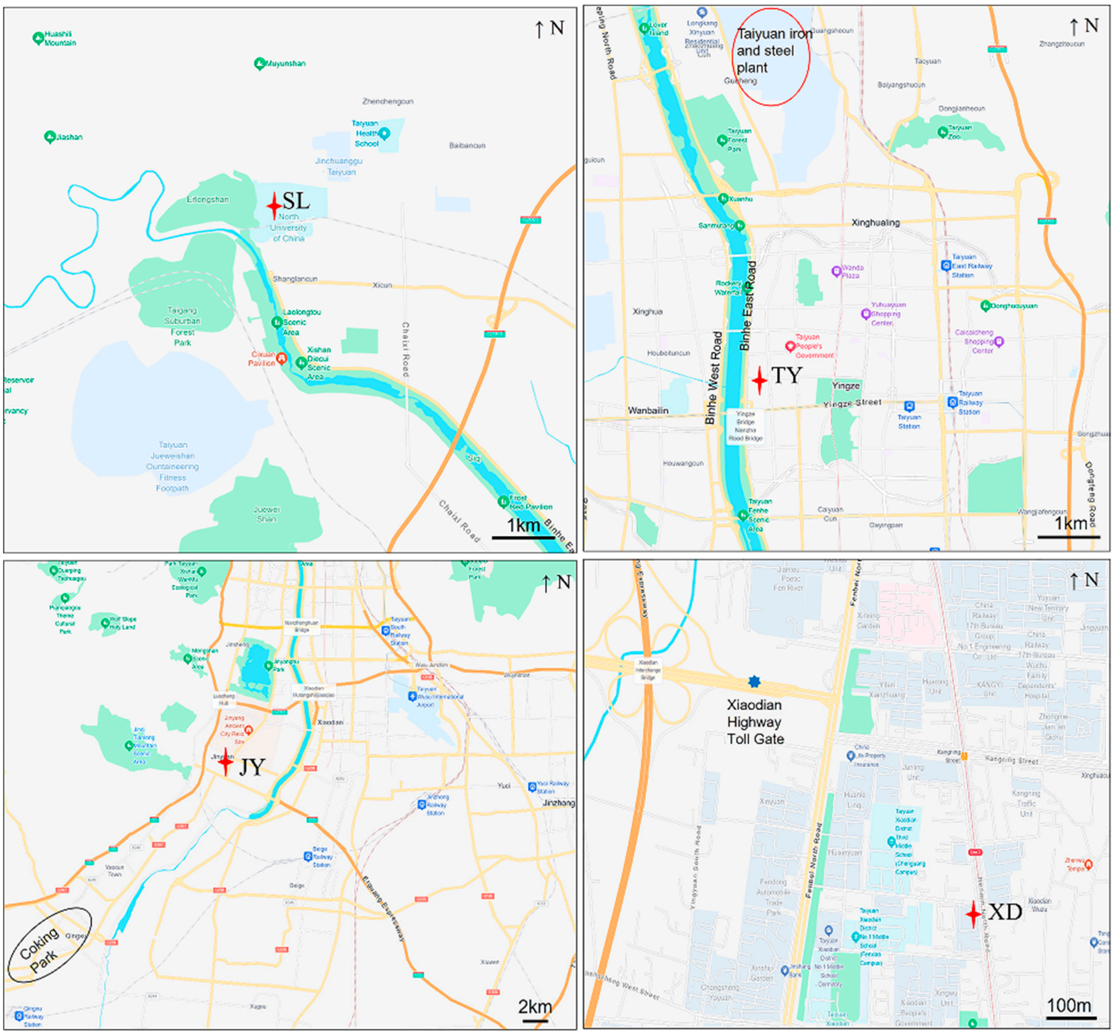

Figure S1. The detailed information on the surroundings of the sampling sites

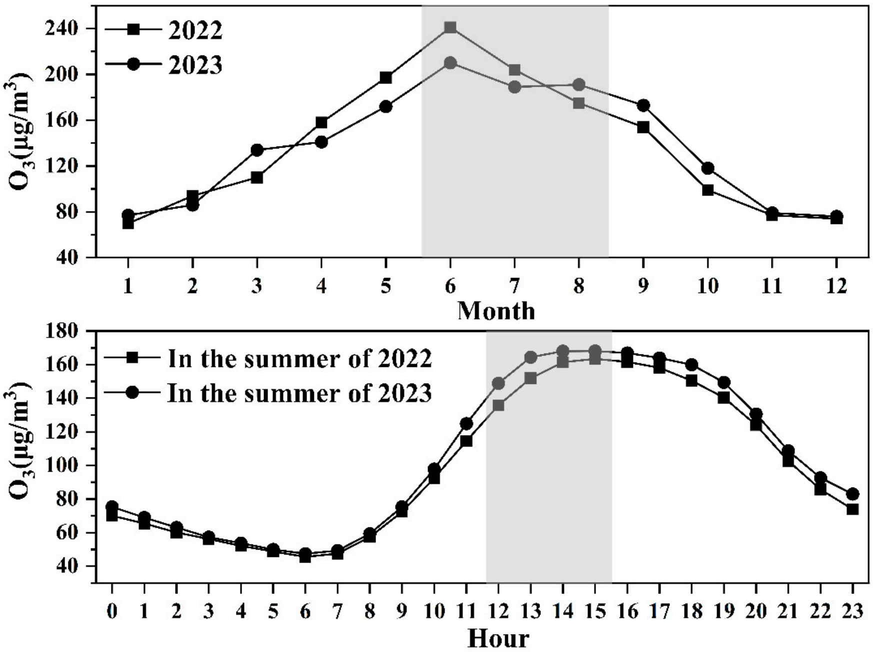

Figure S2. Monthly and diurnal variations of O_3 in Taiyuan in 2022 and 2023

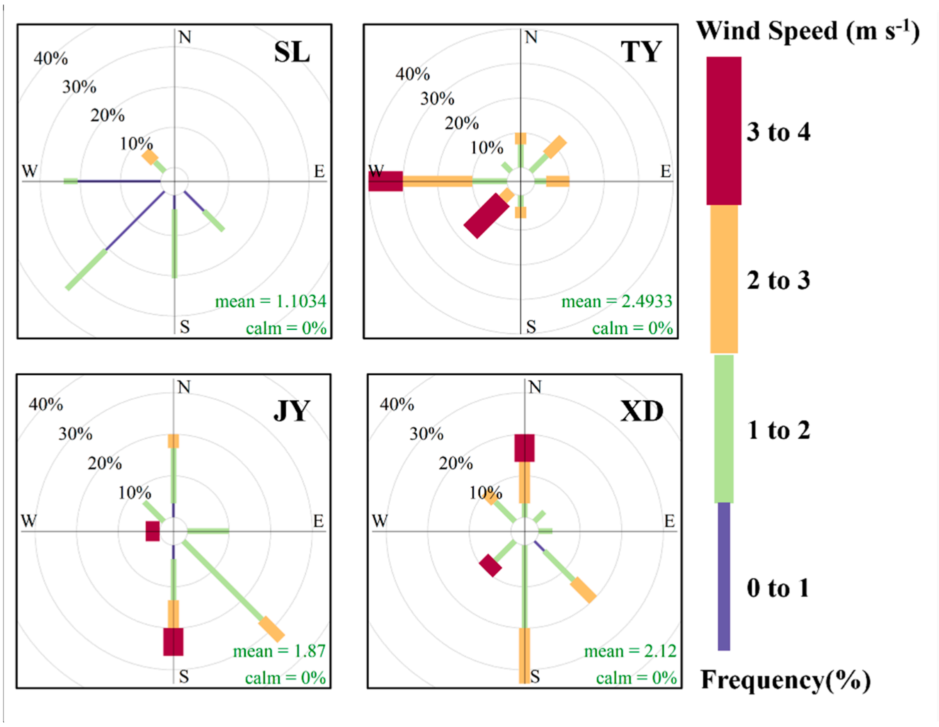

Figure S3. Wind rose diagram during the sampling periods at SL, TY, JY and XD

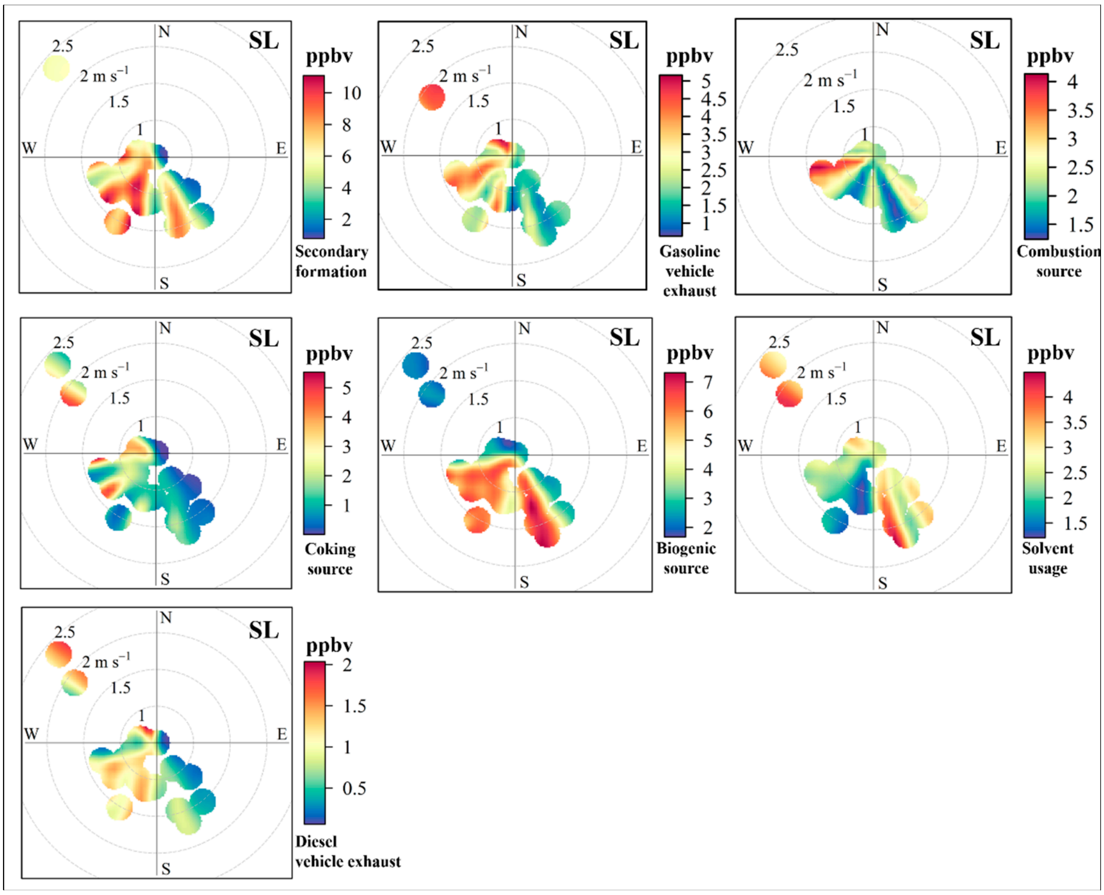

Figure S4. Wind plots for seven pollution source concentrations at SL

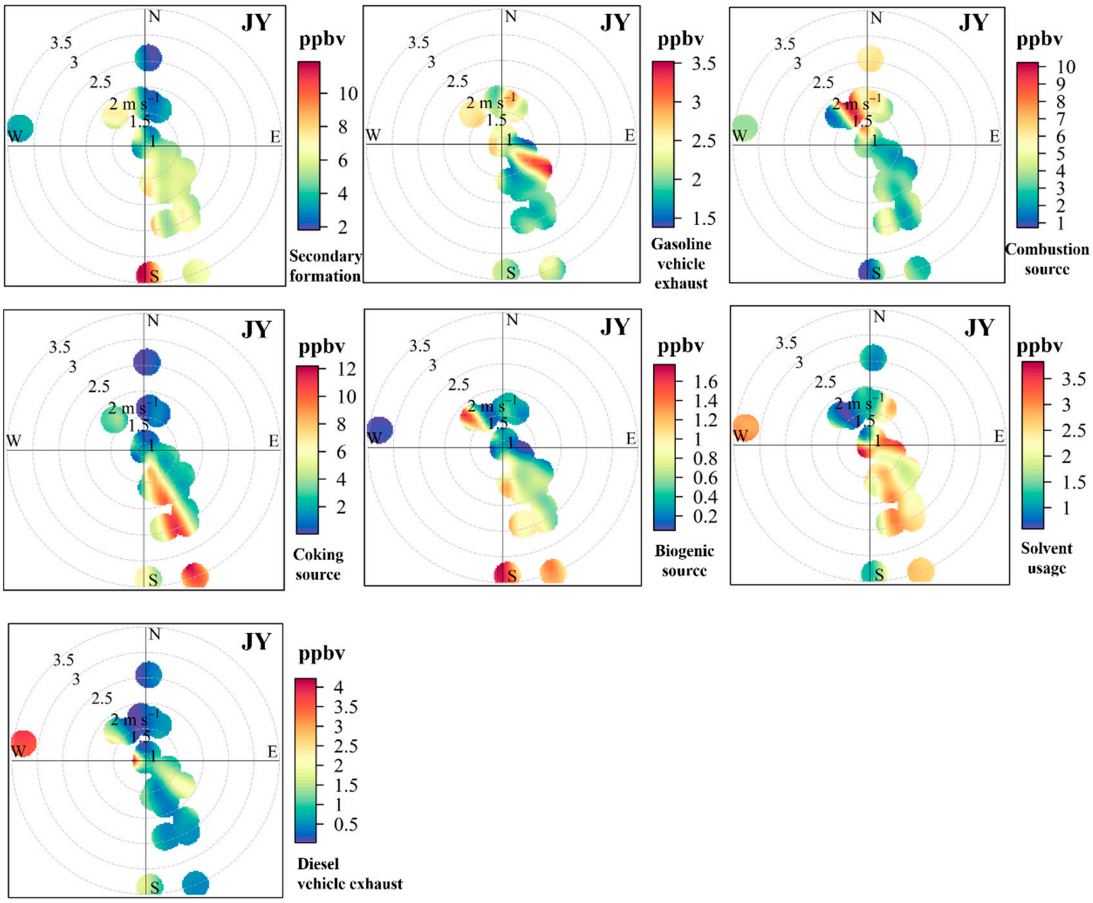

Figure S5. Wind plots for seven pollution source concentrations at JY

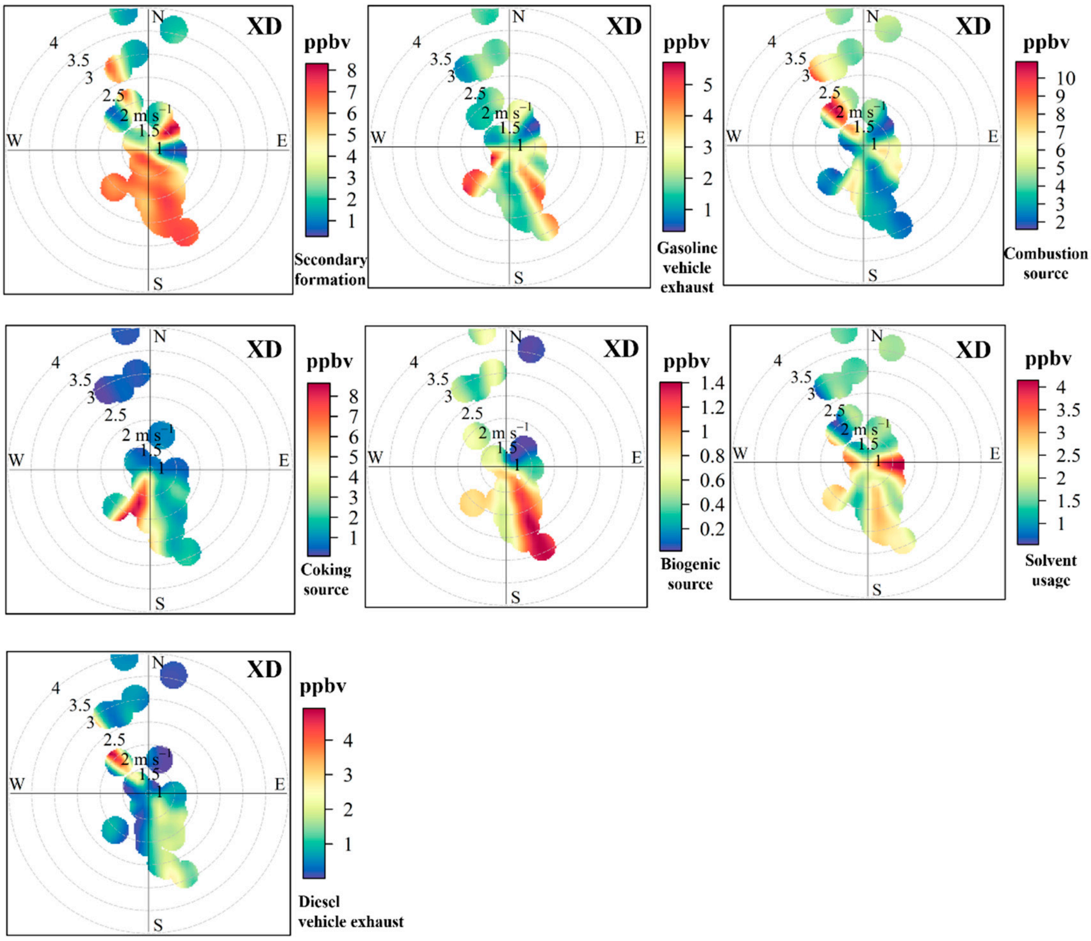

Figure S6. Wind plots for seven pollution source concentrations at XD

Table S1. List of method detection limits in 2022 and 2023 (ppbv)

Group	Species	2022	2023
**Alkanes**	Ethane	0.047	0.067
	Propane	0.061	0.054
	Isobutane	0.076	0.047
	n-butane	0.075	0.047
	Isopentane	0.058	0.048
	n-pentane	0.063	0.050
	2,2-Dimethylbutae	0.053	0.048
	Cyclopentane	0.057	0.053
	2,3-Dimethylbutane	0.050	0.058
	2-Methylpentane	0.060	0.098
	3-Methylpentane	0.052	0.051
	n-hexane	0.049	0.054
	2,4-Dimethylpentane	0.062	0.056
	Methylcyclopentane	0.049	0.052
	Cyclohexane	0.050	0.052
	2-Methylhexane	0.049	0.056
	2,3-Dimethylpentane	0.057	0.057
	3-Methylhexane	0.050	0.054
	2,2,4-Trimethylpentane	0.053	0.056
	n-heptane	0.047	0.055
	Methylcyclohexane	0.045	0.052
	2,3,4-Trimethylpentane	0.043	0.056
	2-Methylheptane	0.042	0.056
	3-Methylheptane	0.048	0.059
	n-octane	0.038	0.053
	n-Nonane	0.041	0.046
	n-Decane	0.072	0.043
	n-undecane	0.044	0.040
	n-dodecane	0.033	0.036
**Alkenes**	Ethylene	0.031	0.060
	Propylene	0.054	0.061
	1-Butene	0.094	0.046
	Cis-2-butene	0.068	0.048
	Trans-2-butene	0.067	0.046
	1-Pentene	0.057	0.056
	Trans-2-pentene	0.054	0.048
	Isoprene	0.051	0.063
	Cis-2-pentene	0.053	0.051
	1-Hexene	0.045	0.049
**Aromatics**	Benzene	0.049	0.052
	Toluene	0.047	0.058

	Ethylbenzene	0.044	0.050
	m,p-xylene	0.023	0.054
	Styrene	0.043	0.051
	o-xylene	0.050	0.055
	Isopropylbenzene	0.040	0.043
	n-propylbenzene	0.070	0.043
	o-ethyltoluene	0.038	0.052
	m-ethyltoluene	0.074	0.046
	1,3,5-Trimethylbenzene	0.052	0.057
	p-ethyltoluene	0.077	0.049
	1,2,4-Trimethylbenzene	0.035	0.046
	1,2,3-Trimethylbenzene	0.047	0.052
	m-diethylbenzene	0.058	0.039
	p-diethylbenzene	0.056	0.041
**Alkyne**	Acetylene	0.047	0.047
**OVOCs**	Formaldehyde	0.079	0.050
	Acetaldehyde	0.065	0.040
	Acetone	0.062	0.012
	Acrolein	0.104	0.114
	Propionaldehyde	0.081	0.086
	Crotonaldehyde	0.067	0.074
	Butanone	0.082	0.105
	MACR	0.106	0.063
	Butyraldehyde	0.142	0.089
	Benzaldehyde	0.110	0.088
	Valeraldehyde	0.061	0.058
	m/p-Tolualdehyde	0.057	0.157
	Hexaldehyde	0.076	0.095
	MTBE	0.041	0.047

Table S2 The correlation coefficients (R^2) between observed and modeled values for each VOC species by PMF

VOC species	R^2
Ethylene	0.98
1-Butene	0.50
Isoprene	0.99
Benzene	0.88
Toluene	0.64
Ethylbenzene	0.70
m,p-xylene	0.56
o-xylene	0.60
Ethane	0.88
Propane	0.85
Isobutane	0.74
n-butane	0.92
Isopentane	0.93
n-pentane	0.90
3-Methylpentane	0.68
n-Decane	0.75
n-undecane	0.89
n-dodecane	0.99
Acetylene	0.75
Formaldehyde	0.84
Acetaldehyde	0.84
Acetone	0.71
MACR	0.63
MTBE	0.94

Table S3 Summary of PMF error estimation diagnostics from BS

BS Mapping (r≥0.6)	Secondary formation	Gasoline vehicle exhaust	Combustion source	Coking source	Biogenic source	Solvent usage	Diesel vehicle exhaust	Unmapped
Factor 1	100	0	0	0	0	0	0	0
Factor 2	0	100	00	0	0	0	0	0
Factor 3	1	6	85	0	0	8	0	0
Factor 4	0	0	0	100		0	0	0
Factor 5	0	0	0	0	100	0	0	0
Factor 6	0	0	0	4	0	96	0	0
Factor 7	0	0	0	0	0	0	100	0

Table S4 Concentration levels of VOCs in Taiyuan (ppbv)

Group	Species	SL	TY	JY	XD	Average
		Mean ± SD	Mean ± SD	Mean ± SD	Mean ± SD	Mean ± SD
**Alkanes**	Ethane	1.63±0.95	1.75±0.87	2.07±1.36	1.72±0.97	1.79±0.19
	Propane	0.53±0.37	0.72±0.39	0.89±0.55	0.70±0.37	0.71±0.15
	Isobutane	0.16±0.09	0.28±0.16	0.21±0.1	0.22±0.1	0.22±0.05
	n-butane	0.26±0.16	0.4±0.20	0.39±0.21	0.34±0.16	0.35±0.07
	Isopentane	0.18±0.12	0.35±0.19	0.21±0.12	0.24±0.2	0.24±0.07
	n-pentane	0.12±0.07	0.19±0.11	0.14±0.08	0.14±0.09	0.15±0.03
	2,2-Dimethylbutae	0.01±0.01	0.01±0.01	0.01±0.01	0.01±0.01	0.01±0.00
	Cyclopentane	0.02±0.01	0.03±0.02	0.02±0.01	0.02±0.02	0.02±0.00
	2,3-Dimethylbutane	0.01±0.01	0.02±0.01	0.01±0.01	0.01±0.01	0.01±0.00
	2-Methylpentane	0.01±0.01	0.01±0.01	0.01±0.01	0.01±0.01	0.01±0.00
	3-Methylpentane	0.02±0.02	0.04±0.03	0.03±0.02	0.03±0.03	0.03±0.01
	n-hexane	0.02±0.02	0.04±0.04	0.02±0.01	0.03±0.02	0.03±0.01
	2,4-Dimethylpentane	0.03±0.04	0.04±0.06	0.03±0.04	0.03±0.04	0.03±0.01
	Methylcyclopentane	0.02±0.01	0.02±0.01	0.02±0.01	0.03±0.03	0.02±0.00
	Cyclohexane	0.05±0.02	0.05±0.01	0.05±0.01	0.05±0.01	0.05±0.00
	2-Methylhexane	0.01±0.01	0.02±0.01	0.01±0.01	0.01±0.01	0.01±0.00
	2,3-Dimethylpentane	0.01±0.01	0.01±0.01	0.02±0.02	0.01±0.01	0.01±0.00
	3-Methylhexane	0.01±0.01	0.02±0.01	0.01±0.01	0.01±0.01	0.01±0.00
	2,2,4-Trimethylpentane	0.02±0.03	0.03±0.07	0.02±0.03	0.02±0.02	0.03±0.01
	n-heptane	0.01±0.01	0.01±0.01	0.01±0.01	0.01±0.01	0.01±0.00
	Methylcyclohexane	0.01±0.01	0.01±0.01	0.01±0.01	0.01±0.01	0.01±0.00
	2,3,4-Trimethylpentane	0.01±0.01	0.01±0.01	0.01±0.01	0.01±0.01	0.01±0.00
	2-Methylheptane	0.01±0.01	0.01±0.01	0.01±0.01	0.01±0.01	0.01±0.00
	3-Methylheptane	0.01±0.01	0.02±0.01	0.02±0.01	0.01±0.01	0.01±0.00
	n-octane	0.02±0.01	0.03±0.05	0.02±0.02	0.02±0.01	0.02±0.00
	n-Nonane	0.03±0.02	0.03±0.03	0.02±0.01	0.02±0.01	0.02±0.00
	n-Decane	0.04±0.03	0.04±0.03	0.03±0.02	0.04±0.03	0.04±0.00
	n-undecane	0.07±0.05	0.08±0.06	0.08±0.06	0.09±0.08	0.08±0.01
	n-dodecane	0.26±0.27	0.28±0.29	0.28±0.31	0.38±0.53	0.30±0.05
**Alkenes**	Ethylene	0.42±0.40	0.50±0.46	0.71±1.03	0.45±0.55	0.52±0.13
	Propylene	0.01±0.02	0.00±0.01	0.01±0.01	0.00±0.01	0.01±0.00
	1-Butene	0.09±0.05	0.10±0.04	0.09±0.04	0.09±0.04	0.09±0.00

	Cis-2-butene	0.01±0.01	0.01±0.01	0.01±0.01	0.01±0.01	0.01±0.00
	Trans-2-butene	0.01±0.01	0.01±0.01	0.01±0.01	0.01±0.01	0.01±0.00
	1-Pentene	0.35±0.35	0.27±0.28	0.35±0.40	0.25±0.25	0.30±0.05
	Trans-2-pentene	0.01±0.01	0.01±0.01	0.01±0.01	0.01±0.01	0.01±0.00
	Isoprene	1.57±1.08	0.42±0.25	0.28±0.21	0.30±0.19	0.64±0.62
	Cis-2-pentene	0.01±0.01	0.01±0.01	0.01±0.01	0.01±0.01	0.01±0.00
	1-Hexene	0.02±0.03	0.01±0.01	0.02±0.06	0.01±0.01	0.02±0.00
**Aromatics**	Benzene	0.18±0.19	0.22±0.21	0.49±1.01	0.28±0.45	0.29±0.14
	Toluene	0.07±0.06	0.10±0.09	0.09±0.10	0.08±0.07	0.09±0.01
	Ethylbenzene	0.03±0.02	0.03±0.02	0.03±0.02	0.03±0.02	0.03±0.00
	m,p-xylene	0.05±0.05	0.06±0.03	0.05±0.04	0.05±0.04	0.05±0.00
	Styrene	0.02±0.02	0.02±0.02	0.02±0.02	0.02±0.02	0.02±0.00
	o-xylene	0.03±0.02	0.03±0.02	0.03±0.02	0.03±0.02	0.03±0.00
	Isopropylbenzene	0.01±0.01	0.01±0.01	0.01±0.01	0.01±0.01	0.01±0.00
	n-propylbenzene	0.01±0.01	0.02±0.01	0.02±0.01	0.01±0.01	0.02±0.00
	o-ethyltoluene	0.01±0.01	0.02±0.01	0.02±0.01	0.01±0.01	0.02±0.00
	m-ethyltoluene	0.02±0.02	0.02±0.01	0.02±0.01	0.02±0.01	0.02±0.00
	1,3,5-Trimethylbenzene	0.01±0.02	0.01±0.02	0.01±0.02	0.01±0.02	0.01±0.00
	p-ethyltoluene	0.05±0.03	0.05±0.03	0.05±0.04	0.05±0.03	0.05±0.00
	1,2,4-Trimethylbenzene	0.02±0.02	0.03±0.02	0.02±0.02	0.02±0.02	0.02±0.00
	1,2,3-Trimethylbenzene	0.02±0.03	0.03±0.03	0.02±0.03	0.02±0.03	0.02±0.00
	m-diethylbenzene	0.02±0.02	0.02±0.02	0.02±0.02	0.02±0.02	0.02±0.00
	p-diethylbenzene	0.02±0.03	0.02±0.03	0.02±0.03	0.02±0.03	0.02±0.00
**Alkyne**	Acetylene	0.57±0.38	0.74±0.48	0.69±0.42	0.62±0.37	0.66±0.07
**OVOCs**	Formaldehyde	7.06±3.18	6.58±2.64	7.84±5.19	6.51±2.55	7.00±0.61
	Acetaldehyde	2.36±0.94	1.94±0.63	1.80±0.71	2.00±1.02	2.03±0.24
	Acetone	3.48±0.94	2.97±0.70	2.97±0.76	2.82±0.57	3.06±0.29
	Acrolein	0.07±0.04	0.07±0.04	0.07±0.05	0.07±0.05	0.07±0.00
	Propionaldehyde	0.23±0.08	0.16±0.05	0.15±0.08	0.15±0.06	0.17±0.04
	Crotonaldehyde	0.07±0.02	0.07±0.02	0.07±0.02	0.06±0.02	0.07±0.00
	Butanone	0.45±0.24	0.41±0.22	0.37±0.22	0.42±0.20	0.41±0.03
	MACR	0.26±0.10	0.15±0.07	0.19±0.08	0.16±0.06	0.19±0.05
	Butyraldehyde	0.12±0.08	0.09±0.05	0.08±0.07	0.08±0.06	0.09±0.02
	Benzaldehyde	0.11±0.06	0.09±0.04	0.09±0.03	0.09±0.04	0.09±0.01
	Valeraldehyde	0.07±0.04	0.06±0.04	0.06±0.04	0.05±0.03	0.06±0.01

	m/p-Tolualdehyde	0.26±0.11	0.21±0.11	0.24±0.08	0.23±0.10	0.23±0.02
	Hexaldehyde	0.1±00.04	0.10±0.03	0.10±0.07	0.10±0.05	0.10±0.00
	MTBE	0.03±0.01	0.04±0.03	0.08±0.05	0.07±0.05	0.05±0.02

Table S5 The top 10 VOCs ranked according to calculated OFP

**Site**	**Species**	**OFP (ppbv)**	**%**	**Site**	**Species**	**OFP (ppbv)**	**%**
SL	Formaldehyde	66.8	55.8	TY	Formaldehyde	62.2	62.0
	Isoprene	16.6	13.9		Acetaldehyde	12.7	12.7
	Acetaldehyde	15.5	12.9		Ethylene	4.5	4.5
	Ethylene	3.7	3.1		Isoprene	4.5	4.5
	1-Pentene	2.5	2.1		1-Pentene	1.9	1.9
	Propionaldehyde	1.6	1.3		Propionaldehyde	1.2	1.2
	MACR	1.6	1.3		Acetone	1.1	1.1
	Acetone	1.3	1.0		1-Butene	0.9	0.9
	1-Butene	0.9	0.7		MACR	0.9	0.9
	Butyraldehyde	0.7	0.6		Acetylene	0.7	0.7
Sum	—	111.1	92.9		—	90.6	90.2
JY	Formaldehyde	74.2	66.2	XD	Formaldehyde	61.6	63.5
	Acetaldehyde	11.8	10.5		Acetaldehyde	13.1	13.5
	Ethylene	6.4	5.7		Ethylene	4.0	4.1
	Isoprene	3.0	2.7		Isoprene	3.2	3.2
	1-Pentene	2.5	2.3		1-Pentene	1.8	1.9
	MACR	1.1	1.0		Propionaldehyde	1.1	1.1
	Acetone	1.1	1.0		Acetone	1.0	1.0
	Propionaldehyde	1.0	0.9		MACR	1.0	1.0
	1-Butene	0.8	0.7		1-Butene	0.9	0.9
	Acetylene	0.7	0.6		Butanone	0.6	0.6
Sum	—	102.5	91.6		—	88.2	90.9
